# Lung Cancer—Epidemiology, Pathogenesis, Treatment and Molecular Aspect (Review of Literature)

**DOI:** 10.3390/ijms26052049

**Published:** 2025-02-26

**Authors:** Beata Smolarz, Honorata Łukasiewicz, Dariusz Samulak, Ewa Piekarska, Radosław Kołaciński, Hanna Romanowicz

**Affiliations:** 1 Laboratory of Cancer Genetics, Department of Pathology, Polish Mother’s Memorial Hospital Research Institute, 93-338 Lodz, Poland; hanna-romanowicz@wp.pl; 2 Faculty of Medicine and Health Sciences, Department of Nursing, The President Stanisław Wojciechowski Calisia University, 62-800 Kalisz, Poland; sekretariat@szpital.kalisz.pl; 3 Department of Obstetrics and Gynecology and Gynecological Oncology, Regional Hospital in Kalisz, 62-800 Kalisz, Poland; samulakd@wp.pl; 4 Department of Obstetrics, The President Stanisław Wojciechowski Calisia University, 62-800 Kalisz, Poland; 5 Regional Hospital in Kalisz, 62-800 Kalisz, Poland; m.smol@wp.pl (E.P.); r.kolacinski@szpital.kalisz.pl (R.K.)

**Keywords:** lung cancer, pathogenesis, treatment, genetics

## Abstract

Lung cancer is one of the most common malignant cancers in most countries and is the leading cause of death among cancer diseases worldwide. Despite constant progress in diagnosis and therapy, survival rates of patients diagnosed with lung cancer remain unsatisfactory. Numerous epidemiological and experimental studies conducted as early as the 1970s confirm that the most important risk factor for the development of lung cancer is long-term smoking, which remains valid to this day. In the paper, the authors present the latest data on the epidemiology, pathogenesis, treatment and molecular aspects of this cancer. In the last decade, many molecular alterations that are effective in the development of lung cancer have been discovered. In adenocarcinoma, tyrosine kinase inhibitors were developed for EGFR mutations and ALK and ROS1 translocations and were approved for use in the treatment of advanced stage adenocarcinomas. In the case of squamous cell carcinoma, the evaluation of these mutations is not yet being used in clinical practice. In addition, there are ongoing studies concerning many potential therapeutic molecular targets, such as ROS, MET, FGFR, DDR-2 and RET. Constant progress in diagnostic and therapeutic methods gives rise to hopes for an improved prognosis in patients with lung cancer.

## 1. Introduction

Malignant lung cancer is still a current clinical problem, posing a real threat to the entire world population [1,2]. In order to improve the statistics of early detection of lung cancer, numerous screening projects have been introduced in recent years, which at the were simultaneously intended to translate into an improvement in the prognosis of the disease [3,4,5]. Knowing that the risk of developing lung cancer increases in people who smoke for a long time and intensively, in the years 2002–2004 in the United States, the National Lung Screening Trial (NLST) program was implemented [6]. This study included patients from the highest risk groups, i.e., people between 55 and 74 years of age who were active smokers with a history greater than or equal to 30 pack-years. Qualified patients underwent screening using low-dose computed tomography (LDCT), performed at one-year intervals. The results of the study showed a reduction in the relative risk of death by about 20% in people examined using LDCT, compared to the group monitored by conventional X-rays. However, this was associated with a high percentage of false positives and unjustified thoracic surgical interventions [7,8]. Given the lack of reliable, population-wide screening, the efforts of scientists have been focused for several years on understanding the molecular mechanisms useful in the effective selection of optimal therapy for patients at various stages of the disease [9,10]. Knowledge of gene polymorphisms in lung tumors and their clinical usefulness is evolving. Initially, it was thought that these studies would make it possible to predict the response to the use of classic cytostatics such as cisplatin. Currently, more and more data indicate that the determination of tumor polymorphisms may be an element belonging to the panel of prognostic markers [11,12].

## 2. History of Lung Cancer

Lung cancer is a medical term that was introduced to clinical oncology at the beginning of the twentieth century. The nomenclature of lung tumors has evolved over the years, but it was crucial for the history of medicine to learn about the causative factors causing this disease entity [13,14].

In 1879, Harting and Hesse confirmed, on the basis of numerous autopsy examinations, earlier reports that miners were a group particularly predisposed to the occurrence of proliferative lesions in the lung tissue [15]. Thanks to the above-cited research, the first potential risk factor for lung cancer, which was occupational, long-term exposure to mine dust, was identified for the first time. The list of factors potentially triggering lung cancer has gradually grown. Subsequently, it was supplemented with exposure to ionizing radiation, the source of which was radon present in the mines [16]. A significant increase in the incidence and mortality of lung cancer observed since the 1930s, resulting from the spread of smoking, has made it possible to identify the most dangerous risk factor today, which is nicotine smoke [13,17,18]. It should be emphasized that the first reliable reports in the scientific press about the probable effect of smoking on increasing the risk of lung cancer date back to 1939. The research of Ochsner and DeBakey, presented at that time, conducted on the basis of observations of several dozen patients with lung cancer, confirmed the increase in the incidence of this cancer observed in the first half of the last century in patients with heavy smoking [19,20,21]. In 1950, retrospective observations from both Britain and the United States were published almost simultaneously and contained scientific evidence and presented consistent conclusions about the groundbreaking importance of exposure to tobacco smoke.

Previous observations that smoking is associated with an increase in the incidence and mortality of lung cancer have been confirmed [22,23].

## 3. Epidemiology of Lung Cancer

Lung cancer is currently the most commonly diagnosed malignant tumor and is the most common cause of death caused by cancer in the world [24]. Lung cancer has the leading cancer incidence in the world. The global cancer statistics by world regions for the year 2022 based on updated estimates from the International Agency for Research on Cancer (IARC) are as follows. In 2022, there were nearly 20 million new cases of cancer (including non-melanoma skin cancers [NMSC]) and 9.7 million cancer deaths (including NMSCs) [25]. It is estimated that about one in five men or women will develop cancer during their lifetime. About one in nine men and one in 12 women die from cancer. Lung cancer was the most commonly diagnosed cancer in 2022. It concerned 2.5 million new cases, i.e., it accounted for every eighth cancer in the world (12.4% of all cancers in the world). The next most common cancers were breast cancers in women (11.6%), colorectal cancer (9.6%), prostate cancer (7.3%) and stomach cancer (4.9%). Lung cancer was also the leading cause of cancer deaths, with an estimated 1.8 million deaths (18.7%). The next places were occupied by colorectal cancer (9.3%), liver cancer (7.8%), breast cancer in women (6.9%) and stomach cancer (6.8%). Breast cancer and lung cancer were the most common cancers in women and men, respectively (both cases and deaths).

Incidence rates (including NMSC) ranged from four to five times in different regions of the world, from more than 500 in Australia/New Zealand (507.9 per 100,000) to less than 100 in West Africa (97.1 per 100,000). Among women, it ranged from over 400 in Australia/New Zealand (410.5 per 100,000) to close to 100 in South Central Asia (103.3 per 100,000) [25].

## 4. Pathogenesis of Lung Cancer

**Figure 1:**
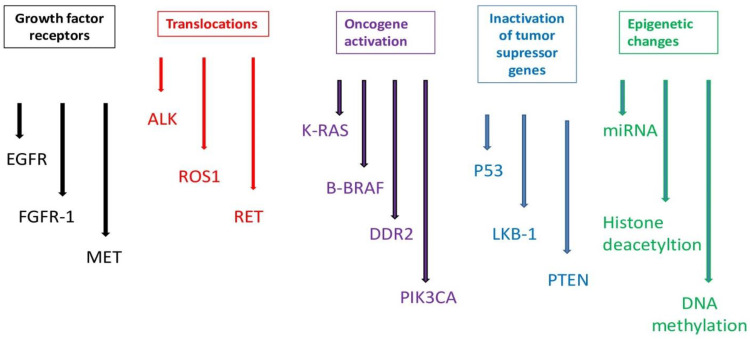
Molecular alterations in lung cancer.

The process of molecular pathogenesis of lung cancer is quite complicated and heterogeneous. Lung cancer can develop as a consequence of several genetic factors and epigenetic changes (for example, point mutations, amplifications, insertions, deletions and translocations). This is particularly associated with the activation of a pathway that promotes growth and inhibition of the tumor suppressor pathways (Figure 1) [26].

**Figure 2:**
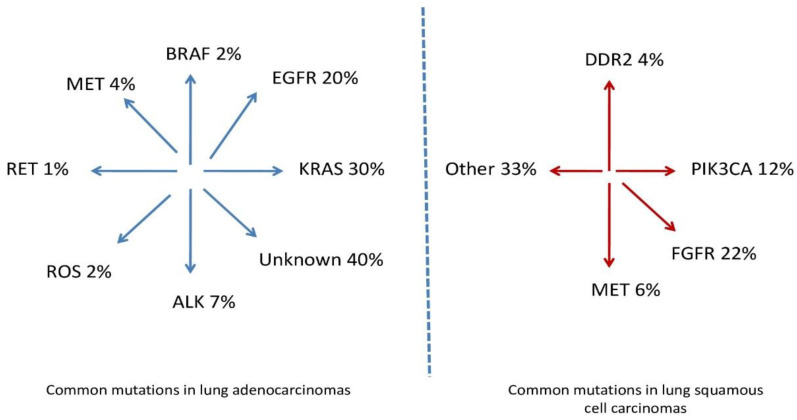
Common mutations in lung adenocarcinomas and common mutations in lung squamous cell carcinomas.

Studies indicate that adenocarcinomas and squamous cell carcinomas, both subtypes of NSCLC, are quite rare and differ in molecular characteristics [27,28]. The precursor mutations and the prevalences of these mutations in NSCLC by histologic subtype are presented in Figure 2.

The molecular basis of lung cancer should be understood as the accumulation of many genetic and epigenetic changes in the nucleus of the cell, which occur over a long period of time [29]. The cancer process is initiated when a given cell breaks out of the control of the mechanisms that determine its division and location. Its cell cycle is similar to that of normal cells, with the difference that this cell does not submit to regulatory mechanisms and becomes insensitive to signals from other cells. Disorders in the expression of genes regulating the cell cycle play a key role in any neoplastic transformation. The initiation and progression of the neoplastic process is influenced by the following:

- Abnormalities in the regulation of the cell cycle;
- Mutations in proto-oncogenes and tumor suppressor genes;
- Disorders of the DNA repair process;
- Increased expression of growth factors and angiogenesis;
- Avoidance of apoptosis (mutations of anti- and pro-apoptotic genes);
- Increased telomerase activity;
- Tissue invasion and metastasis.

The instability of the entire cell genome, which occurs at the beginning of the carcinogenesis process, also plays an important role. It is the result of the gradual accumulation of various genetic abnormalities. This leads to a weakening of the DNA structure and its greater susceptibility to further mutations [30,31]. Disorders of cell cycle regulation concern mutations in proto-oncogenes and tumor suppressor genes. Lack of proliferation inhibition or accelerated proliferation so that the cell is not sensitive to inhibitory signals is the essence of any cancer process. The tumor suppressor genes in which mutations in lung cancer cells are most common include *TP53*, *RB* and *p16* [32]. The proto-oncogenes that most often mutate in lung cancer are the *MYC*, *RAS* and *HER* gene families. The process of *ALK* gene rearrangement is also of great importance [33]. Growth factors are a broad group of peptide compounds produced by various cell types and affect proliferation and differentiation, as well as metastasis and apoptosis. Receptors for them are located on the surfaces of cells and play an important role in transmitting signals from the extracellular space to the inside of the cell. One of the growth factors is epidermal growth factor (EGF), which is most important in NSCLC, together with its epidermal growth factor receptor (EGFR) [34]. Disruption of its function is one of the most important elements in the pathogenesis of lung cancer. Vascularization is a very important element of the structure of all tissues, both normal and pathologically changed. Physiologically, this process is self-limiting, unlike abnormal angiogenesis, wherein endothelial cells divide several hundred times longer and their survival time is much longer. Vascular endothelial growth factor (VEGF) is one of the main and strongest pro-angiogenic factors. In a cancerous tumor, the VEGF-mediated pathway is constantly activated, and the predominance of pro-angiogenic factors over antiangiogenic factors allows tumor growth. A tumor that grows larger than 3 mm in diameter needs its own vascular network to continue to grow [35]. Hence, the moment of acquiring the ability to create its own vessels that nourish the tumor is an extremely important factor in the progression of the cancer process. The mRNA for *VEGF* is present in most cancer cells, including lung cancer. In endothelial cells, a greater expression of VEGFR is observed than of VEGF itself. This suggests that VEGF in tumor cells may have a paracrine effect—secreted from cancer cells, it acts with the help of receptors on endothelial cells and activates them. It has also been proven that VEGF accumulates near the tumor site no more than 0.5 mm from the tumor border [35]. The vessels feeding the tumor have a chaotic arrangement and variable diameter and are fragile and brittle, which may result in one of the clinical symptoms, hemoptysis, which should always increase oncological vigilance in the physician treating such a patient. A decrease in the ability of cells to properly perform apoptosis as a response to stimuli from the surrounding environment is an important factor in the pathogenesis of lung cancer. Apoptosis, also known as programmed, suicidal, active or physiological cell death, is a process that determines the homeostasis of the body.

The molecular basis of lung cancer shows that the accumulation of many genetic abnormalities is needed for this cancer to develop. The rapid development of molecular biology that has been observed in recent years creates opportunities for an in-depth analysis of the changes in the genome that lead to the development of cancer. During carcinogenesis, mutations in the *p53* gene, which is multidirectional, are of key importance. Mutations of other genes (*Rb*, *MYC*, *RAS*, *EGFR*) coexisting with p53 protein dysfunction result in a significantly higher probability of neoplastic transformation. In clinical practice, it is important to search for abnormalities in specific genes that, as molecular predictive factors, can contribute to more targeted and effective cancer therapy and determine a better prognosis. The use of predictive tests in clinical practice with the use of all the markers mentioned in this paper is not yet a routine procedure. However, some of them are a strong point in the diagnostic process in lung cancer and have been inscribed in the algorithm of therapeutic management. These include determining the mutation profile in the *EGFR* gene, on the basis of which the patient is qualified for targeted treatment of EGFR TKI. Determination of rearrangements in the *ALK* gene is also the basis for ALK inhibitor therapy. However, the authors of the study hope that thanks to progress in molecular diagnostics, it will be possible to detect lung cancer at an early, non-invasive stage in the near future, which will create a chance for effective anti-cancer therapy.

## 5. Clinical Description of Lung Cancer

In the case of suspected lung cancer, the classic physical examination consists of a detailed medical history and a thorough assessment of active and passive exposure to tobacco smoke, family history of cancer and occupational exposure to other known carcinogenic agents [36,37]. Physical examination of patients with suspected lung cancer should take the following into account: symptoms associated with bronchial stenosis or obstruction (weakening of the alveolar murmur, localized wheezing over the affected bronchi, bronchial or tracheal murmur). Next, it should be determined whether there are enlarged lymph nodes (especially supraclavicular nodes), as well as symptoms associated with the presence of fluid in the pleural cavity, such as suppression of the tapping sound or weakening of the alveolar murmur [38,39].

Symptoms of the presence of fluid in the pericardial sac and myocardial infiltration are diagnosed on the basis of the following symptoms: enlargement of the heart silhouette, weakening of heart sounds, widening of the jugular veins, low amplitude of arterial pressure and cardiac arrhythmias [40,41].

In patients with advanced cancer, symptoms of superior vena cava syndrome are often observed, i.e., swelling of the face, enlargement of the neck circumference, swelling of the upper limbs, widening of the jugular veins and veins on the chest wall and bruising of the facial skin and mucous membranes [42,43].

Local and generalized clinical symptoms accompanying lung tumor growth are presented in Table 1.

## 6. Diagnosis and Staging of Lung Cancer

Determining the stage of lung cancer includes an assessment of the status of the primary tumor (feature T, tumor), regional lymph nodes (feature N, node) and organs where metastases may be present (feature M, metastasis). In patients qualified for radical treatment, it is necessary to determine the size and location of the primary tumor and its relation to the surrounding anatomical structures (chest wall, pleura, diaphragm, heart, large vessels and esophagus) and the condition of regional lymph nodes. The list of tests used in the assessment of advancement is presented in Table 2. On the basis of the combined assessment of the T, N and M features (Table 3), the clinical stage of NSCLC is determined (Table 4). At the time of diagnosis of NSCLC, the proportion of patients in stages I–II, III and IV is approximately 25%, 35% and 40%, respectively.

Until now, a simplified classification was used in the assessment of the severity of SCLC, which distinguished the stage of limited disease (LD) or extensive disease (ED). A limited disease was defined as a neoplasm that did not exceed one half of the chest, regardless of the involvement of hilar lymph nodes on the side of the lesion and mediastinal and supraclavicular nodes on both sides, not excluding the presence of neoplastic effusion in the pleural cavity on the side of the tumor. The presence of tumor foci outside the mentioned area meant a diagnosis of the stage of extensive disease. Currently, in the SCLC, as in the NSCLC, it is recommended to use the TNM classification [45]. The incidence of SCLC in TNM grades I–III and IV at the time of cancer diagnosis is approximately 35% and 65%, respectively. In patients with lung cancer who underwent excision of the lung parenchyma and lymph nodes, the final stage is determined on the basis of pathomorphological examination of the surgical material. The pathological TNM (pTNM) stage determined in this way is more accurate and reflects the prognosis of patients better than the clinically determined stage (cTNM, clinical TNM) [44,46].

Recommendations:

— Staging assessment of non-small cell and small cell lung cancer should be carried out using the principles and criteria of the current TNM classification (IV, A).
— In the presence of two lesions suspected of primary cancer, a separate staging assessment should be carried out (III, A).
— In patients with lung cancer with features of mediastinal lymph node involvement, pathomorphological confirmation of the nature of the suspicious lesions should be obtained during imaging studies when qualifying for possible resection of the lung parenchyma (IV, B).
— In patients prior to planned radical treatment, pathomorphological confirmation of the possible presence of neoplasm in single suspicious lesions located in other organs detected by imaging tests is recommended if possible (IV, A).
— In patients with lung cancer who underwent excision of the lung parenchyma and lymph nodes, the final stage is determined on the basis of pathomorphological examination of the surgical material (IV, A).

Radiographic imaging of lung cancer varies widely. A suspected lung tumor on conventional chest X-ray (X-ray) in anteroposterior and lateral projections may suggest the presence of a circular shadow.

The presence of a proliferative lesion may also be evidenced by the following: changes in the outline of the lung hilum, airborne disorders of the lung parenchyma in the form of atelectasis, the presence of an infiltrative lesion and pleural effusion [47]. A normal result of a conventional X-ray examination does not exclude cancer, which may be located in areas with limited availability (the top of the lung or the mediastinal area); therefore, a computed tomography (CT) of the chest should be performed in all patients with suspicious symptoms. As a standard, a CT scan should be performed with the use of a contrast agent administered intravenously and additionally include the abdominal cavity up to the level of the adrenal glands. In a CT scan, the image suggesting a malignant lesion may take the form of a solid or partially solid tumor, as well as an image of the so-called milky glass—ULN (ground glass nodule) [48,49]. In special situations, magnetic resonance imaging (MRI) of the chest is performed. This examination can determine the state of tumor infiltration of surrounding structures such as the spine, mediastinum, chest wall and diaphragm [50]. In the presence of a single nodule of an undetermined nature and a diameter of more than 1 cm in the lung parenchyma, positron emission tomography (PET) in combination with CT (PET-CT) may be helpful. This examination facilitates the differentiation of benign and malignant lesions and determines indications for other tests or observations. It is also helpful in assessing the extent of the tumor before further planned surgical treatment or radical irradiation [51,52]. PET-CT also has the highest sensitivity in assessing the condition of the mediastinal lymphatic system and detecting distant metastases. The factor differentiating the neoplastic nature of lesions in PET-KT is the standardized uptake value (SUV), which depends on many variables. Therefore, as is often emphasized in the literature, it is not always reliable to determine the final conclusions on the malignancy of a change based solely on the SUV value, due to the possibility of false positive or false negative results. Taking these data into account, in any case, the results of the PET-KT test should only be considered as an additional test [53,54]. Basic tests in the pathomorphological diagnosis of lung cancer include histological assessment of a specimen taken during endofiberoscopy or cytological assessment of a smear or bronchial lavage. Endobronchial tweezer biopsy remains the gold diagnostic standard, during which numerous samples of tumor or bronchial infiltration can be taken. In recent years, a cryo probe has been used to collect cancerous tissue infiltrating the bronchi, thanks to which it is possible to collect sections as large as 5–8 mm [55].

Since the 1990s, fiberscopes with an ultrasound probe have been used to diagnose extrabronchial lesions or enlarged mediastinal lymph nodes. Bronchofiberoscopic examinations with endobronchial ultrasonography EBUS-TBNA (endobronchial ultrasound-guided transbronchial needle aspiration) or intraesophageal EUS-FNA (endoscopic ultrasound-guided fine needle aspiration) ultrasound can also be performed to assess the advancement of cancer [56,57]. In the case of peripheral lesions, the diagnosis is usually made on the basis of cytological or histological assessment of the material obtained by biopsy through the chest wall. Current standards of conduct emphasize that it is necessary to strive to collect enough material to determine the type and subtype of cancer and perform molecular tests. Therefore, core needle transtortorial biopsy is the preferred procedure for lesions not available in endoscopic examinations [58]. More invasive ways to obtain tissue material include cervical mediastinoscopy, parasternal mediastinotomy, surgical biopsy of peripheral lymph nodes, diagnostic videothoracoscopy or exploratory thoracotomy—a procedure performed after exhausting all other options [59]. Cytological assessment of sputum or exudative fluid present in advanced stages of the disease is a low-sensitivity test used only when bronchoscopy or chest biopsy cannot be performed [60].

## 7. Pathomorphology and Molecular Diagnostics of Lung Cancer

Primary lung cancer is a cancer that originates from epithelial cells. The most common (about 85% of all diagnoses) are non-small cell lung cancers (NSCLCs). The majority of NSCLCs are adenocarcinomas and squamous cell carcinomas (the incidence of adenocarcinoma has been increasing recently). The incidence of diagnosis of large cell lung cancer has decreased to approximately 2% since the introduction of immunohistochemistry (IHC). Small cell lung cancer (SCLC) currently accounts for about 13% of all primary lung cancers and differs from other histological types in many biological and clinical features (high proliferation rate, short doubling time of tumor mass, pronounced tendency for early metastasis, chemosensitivity and relative radiosensitivity) [61]. Other histological types account for less than 1% of all primary lung cancer diagnoses. Lung cancer develops centrally in the area of the large bronchi (the so-called hilar lesion) or peripherally. Glandular carcinomas are more common in the peripheral parts of the lungs, and squamous cell carcinomas are more common in the parahilar. Metastases most often appear in regional lymph nodes (followed by liver, brain, second lung, bones, adrenal glands, subcutaneous tissue and bone marrow). Metastases can also form in distant organs without involving regional lymph nodes. Lung cancer can also spread locally by infiltrating the mediastinal structures, diaphragm, pleura and chest wall and filling the surrounding air spaces. Classification of epithelial lung cancers according to the World Health Organization (WHO, World Health Organization) [62] (Table 5) introduced changes, the most important of which is the introduction of the following:

— Rules for handling small specimens and cytological material (especially in advanced forms of NSCLC);
— A new division of adenocarcinomas and squamous cell carcinomas;
— Rhe need to use IHC and genetic tests in pathomorphological diagnostics in order to individualize treatment;
— Diagnosis of large cell carcinoma and other—rare—NSCLCs only in postoperative material;
— Classification in one group of cancers with features of neuroendocrine activity. The classification also presents new rules for determining the degree of differentiation of adenocarcinomas of the lung (grading), and in the group of neuroendocrine tumors, carcinoids are classified as neuroendocrine tumors, while small cell and large cell neuroendocrine carcinomas are classified as neuroendocrine carcinomas.

The range of procedures used in pathomorphological diagnostics depends on the histological type of cancer and the stage of the disease. The management of preoperative material and small specimens and cytological material (cytoblocks) in patients with inoperable NSCLC requires determination of the type of cancer and, in certain cases, testing of predictive factors to make a decision on the appropriate therapy. It is necessary for pathologists to cooperate closely with the doctors ordering the examination and planning treatment, and rational use of the material submitted for pathomorphological examination is needed as well. Determination of NSCLC type is based on morphological criteria found in standard hematoxylin and eosin (H + E) staining and additional histochemical tests for mucus in cancer cells and IHC, using markers useful in the differentiation of adenocarcinoma (TTF1, thyroid transcription factor) and squamous cell (p40). In the case of an ambiguous histological picture and the inability to determine the type of NSCLC based on tumor morphological features, IHC tests and neuroendocrine indices, the diagnosis of unspecified cancer (NOS) can be established. However, the share of such diagnoses should not exceed 10% of all NSCLC diagnoses. The rate of NOS diagnoses can be reduced due to greater availability of tissue material, which allows for a complete histological diagnosis [62]. Ambiguous histological picture and the expression of markers of glandular differentiation in IHC justify the diagnosis of NSCLC consistent with adenocarcinoma (favor adenocarcinoma), and in the case of immunophenotype of squamous cell carcinoma, NSCLC corresponding to squamous cell carcinoma is diagnosed (non-small-cell lung cancer—favor squamous-cell carcinoma) [62]. Determination of neuroendocrine markers (MNEs) is indicated only when morphological features suggestive of neuroendocrine differentiation are found (routine is not recommended, as 10–20% of all NSCLCs express one of the MNEs, and it has no effect on the management of the cancer) [62]. Pathomorphological diagnosis of the surgical material requires determination of the type and subtype as well as the degree of differentiation of the cancer, the presence of prognostic factors (e.g., embolism of tumor cells in blood or lymphatic vessels, infiltration of nerve and pleural fiber bands and surrounding air spaces, extent of necrosis) and complete resection and indication of the pathomorphological stage of the disease (pTNM). In adenocarcinomas, it is necessary to determine each type of morphological weave found in the tumor, which is important for determining the degree of cancer differentiation (G) [63]. The histological classification of NSCLC is supplemented by the classification according to the degree of differentiation (histological malignancy—G, grade: GX—no differentiation determination, G1—high differentiation, G2—moderate differentiation, G3—low differentiation, G4—undifferentiated cancer). However, the degree of histological malignancy is of limited importance in the choice of treatment method [62]. In patients with advanced non-squamous cell carcinoma, it is necessary to evaluate the EGFR and ALK genes and ROS1 in order to detect their disorders [64,65,66]. The presence of mutations in the EGFR gene or translocation of the ALK and ROS1 genes is a predictive factor and the basis for qualification for targeted treatment with epidermal growth factor receptor (EGFR) tyrosine kinase inhibitors and ALK or ROS1. EGFR and KRAS mutations and ALK and ROS1 translocations are almost always mutually exclusive [64]. Expanding the panel of tested predictive markers to include the detection of disorders of the BRAF, MET, RET, NTRK, HER2 and KRAS genes will be associated with the introduction of new drugs targeting these molecular targets. Diagnostics of predictive biomarkers are currently also recommended in patients with squamous cell carcinoma, which is especially true for young non-smokers, patients diagnosed on the basis of scant biopsy material and patients with mixed NSCLC [65,66]. Gene assessment can be performed using tissue material or, if a sufficient number of cells in the preparation is confirmed, cytological examination (cytological material embedded in a paraffin block is preferred). If immune checkpoint inhibitors are planned, assessment of the expression of programmed death ligand 1 (PD-L1) in tissue or, in the absence of it, in cytological material (cytoblock) should be performed [62,64]. A “liquid” biopsy (usually a peripheral blood plasma test) is a reliable source of circulating free DNA (cfDNA) and, more specifically, the fraction of circulating tumor DNA (ctDNA). Free DNA testing is a recommended alternative to cellular or tissue material assessment for the detection of resistance to first- or second-generation EGFR tyrosine kinase inhibitors (presence of the Thr790Met mutation—commonly referred to as T790M—in the EGFR gene) prior to second-line targeted therapy. Assessment of predictive biomarkers based on circulating DNA analysis prior to first-line treatment is only acceptable in the absence or limited availability of tissue or cellular material [65]. The prognosis of lung cancer patients depends primarily on the primary stage, while the age and sex of patients are less important. The new pathomorphological classification indicates a different clinical course in individual histological subtypes of adenocarcinoma (e.g., better prognosis—wallpapering and papillary subtypes, worse prognosis—small and solid subtypes), which, however, does not affect the choice of treatment method. In patients with advanced cancer, the prognosis depends to the greatest extent on the status of fitness and the degree of weight loss in the period preceding diagnosis. The prognostic significance of activating mutations in the EGFR and ALK genes has not been definitively confirmed, but the presence of these disorders (10–15% and 3–5% of Caucasian patients, respectively) is strongly associated with the activity of appropriate molecularly targeted drugs. The prognosis in the DRP is generally worse than in the NSCLC. In SCLC, in addition to the extent of the tumor, high lactate dehydrogenase (LDH) activity, which is associated with tumor mass, has an adverse prognosis.

### Pathomorphological and Molecular Evaluation

The objectives of pathomorphological examination in the diagnosis of lung cancer include determining the type and histological subtype and extent of the cancer, differentiating primary and secondary lesions, determining the condition of the so-called surgical margins and detecting genetic disorders of significant importance for the choice of systemic treatment [62]. Basic tests in the pathomorphological diagnosis of lung cancer include the following:

— Histological assessment of the specimen collected during bronchofiberoscopy;
— Cytological assessment of the smear or bronchial lavage;
— Histological or cytological evaluation of biopsy material through the chest, bronchial or esophageal wall.

Pathomorphological evaluation should include IHC tests to determine the type and histological subtype of lung cancer and differentiation of primary lung cancers and metastases of other tumors (in practice most often adenocarcinomas). Determination of neuroendocrine markers is indicated only if morphological features of neuroendocrine differentiation are detected [62]. Histological examination of the tissue material should be performed, the assessment of which allows for accurate determination of the type and subtype of the tumor and facilitates the expansion of the scope of molecular tests (particularly important in the case of systemic treatment prior to local treatment and in patients who do not qualify for resection of the lung parenchyma). Appropriate quality material for histological examination includes bronchial specimens (taken with forceps or cryoprobe), fragments obtained by core needle biopsy through the chest wall and, in the case of thicker needles, also endobronchial ultrasound-guided transbronchial needle aspiration (EBUS-TBNA) material. An appropriate amount of good quality and properly secured cytological material (preferably in the form of cytoblocks) also allows for reliable determination of the type and subtype of cancer and molecular tests [62,64]. Depending on the clinical situation and the location of the tumor foci, other methods of obtaining material for histological and cytological examination are also used, such as the following:

— Pap smear examination of pleural effusion and/or needle biopsy of the pleura;
— Needle or surgical biopsy of peripheral lymph nodes;
— Needle biopsy of the metastatic focus;
— Mediastinoskopia;
— Mediastinotomia;
— Thoracoscopy,
— Thoracotomy (after exhaustion of all other options);
— Sputum cytology (a low-sensitivity test, used only when microscopic material cannot be obtained by another method) [66].

Before starting treatment, it is necessary to establish a pathomorphological diagnosis. If there are justified difficulties in obtaining material for testing, with simultaneous clinical and radiological features indicating a very high probability of the presence of cancer, a multidisciplinary council may decide to start treatment without a pathomorphological diagnosis. Modern diagnostics of lung cancer also requires advanced molecular tests. Biomarker assessment can be performed in tissue and cytological material (e.g., in an aspirate obtained by fine needle biopsy through the chest wall or bronchial tubes). It is necessary to confirm a sufficient number of cells in the tested preparation (the tumor should be at least 20%). In the case of cytological material, it is advisable to “sink” the cytological material in a paraffin block [61,64,65]. An alternative to genetic testing using tissue or cytological material is the use of cfDNA present in blood plasma, which comes from dead cancer cells (so-called liquid biopsy), to assess somatic mutations. A negative cfDNA result is not conclusive, and a new biopsy is recommended [67,68]. When qualifying patients diagnosed with adenocarcinoma and unspecified NSCLC for treatment with EGFR tyrosine kinase inhibitors, the presence of clinically relevant primary EGFR gene mutations (activating and responsible for resistance), which de novo occur in 10–15%, respectively, should be assessed in the available material and 1% of patients. Assessment of the EGFR gene in the exon range 18–21 should be performed using a method with high sensitivity and specificity (preferably a test certified for clinical diagnosis) [69,70]. The test must detect mutations in the EGFR gene that occur in at least 1% of known disorders of the gene. It is advisable for laboratories performing genetic testing for lung cancer patients to have two alternative methods of identifying genetic disorders. In the event of failure of treatment with EGFR inhibitors of the first or second generation, it is advisable to re-collect biological material to assess the presence of the T790M mutation in the EGFR gene (mutation of resistance to EGFR tyrosine kinase inhibitors) [64,65]. In patients diagnosed with adenocarcinoma or unspecified NSCLC without activating mutations in the EGFR gene, the status of the ALK and ROS1 genes should be evaluated to detect rearrangements that occur in 3–5% and 1% of patients, respectively [67,68]. The presence of ALK gene rearrangements can be determined directly by fluorescence in situ hybridization (FISH) and new generation sequencing (NGS) or indirectly by using the ALK membrane fusion protein expression test by IHC. In the assessment of ROS1 gene rearrangement, the FISH or NGS method is recommended with the possibility of preselection by expression of the ROS1 fusion protein by IHC. The presence of rearrangements of both genes or corresponding fusion proteins is an indication for the use of ALK or ROS1 tyrosine kinase inhibitors. The NGS method enables simultaneous assessment of the status of many genetic disorders, shortens the time of performing a full range of molecular tests and significantly reduces the use of tissue material. ALK kinase is a receptor tyrosine kinase that is active during organogenesis but is not expressed in the cells of the adult organism (except for the cells of the central nervous system). Rearrangements within the *ALK* gene, leading to the activation of ALK kinase, are found in 2–3% of patients with Caucasian lung cancer. The ALK kinase mutation mainly affects patients with adenocarcinoma (especially young men), and, as in the case of EGFR mutations, a negative association with smoking has been demonstrated [71,72]. The presence of ALK gene rearrangements is a predictive factor of response to ALK tyrosine kinase inhibitors. ROS1 gene rearrangement is found in less than 1% of patients with NSCLC, and its occurrence is a predictor of response to drugs that inhibit ROS1 kinase activity [73]. In about 1–3% of patients with NSCLC, a mutation of the BRAF V600 gene is present, which determines the response to therapy with a combination of a BRAF inhibitor and a MEK inhibitor [74,75]. The mutation of the BRAF gene, like the rearrangement of the *ROS1* gene, is most often detected in patients with adenocarcinoma [76,77]. Due to the complexity and difficulties of interpretation, NGS should be performed only in laboratories with documented experience in this field [65]. Concurrent assessment of clinically relevant biomarkers is recommended on the basis of a single referral [66]. If other molecularly targeted drugs are developed and reimbursed, the scope of research should be extended (e.g., mutations in the BRAF and HER2 genes and rearrangements of the MET, RET and NTRK genes) [66]. High reliability of pathomorphological diagnostics using IHC and molecular biology diagnostics can only be ensured by laboratories with properly documented experience, with a valid European quality control program certificate for all tests, regularly subjected to periodic external quality control and ensuring comprehensiveness and simultaneous execution of analytical procedures.

## 8. Risk Factors

The most important risk factor for lung cancer is smoking, primarily active smoking, but there is objective evidence that passive smoking is also important. Smoking is the cause of 80–90% of lung cancer cases, and the lifetime risk of developing lung cancer among male smokers is about 17%, among smoking women is about 12%, and among non-smokers is about 1.5% [36,78].

The literature emphasizes the higher risk of lung cancer in people with long-term exposure to compounds such as radon, asbestos, polycyclic aromatic hydrocarbons, arsenic, beryllium, cadmium, silicone, vinyl chloride, nickel and chromium compounds and diesel engine exhaust [79,80]. The compounds mentioned above are characterized by a high genotoxic potential, which contributes to the formation of numerous oxidative and nitrative damages. In addition, multidirectional activation of signal transduction pathways is observed, as well as the production of the “cross-talk” phenomenon, which leads to an increase in uncontrolled cellular proliferation [81]. Exposure of the lung area to ionizing radiation applied for other cancers (e.g., early Hodgkin lymphoma or breast cancer) as well as environmental pollution also increases the risk of lung cancer [36]. The occurrence of lung cancer in first-degree relatives is associated with a higher risk of developing the disease than in the general population [82,83].

Lung cancer is most often caused by smoking, but in some patients, it is associated with genetic predisposition, according to a study published by the American Society of Clinical Oncology (ASCO). American specialists have shown that the same predisposition can cause other types of cancer in patients with lung cancer, such as pancreatic cancer, ovarian cancer in women and prostate cancer in men. They warn that the patient’s closest relatives may also be at greater risk of cancer. Experts from the American Society of Clinical Oncology point out that most lung cancers can be associated with cigarette smoking, as well as other environmental factors, such as exposure to asbestos, but in the case of thousands of patients, the development of this disease is driven by inherited genetic factors. Until now, it seemed that adverse genetic changes conducive to lung cancer could be caused by adverse environmental factors, as well as improper lifestyle, including, above all, smoking. However, some people have inherited genetic changes that predispose them more to this cancer. Research by American specialists shows that the detection of inherited genetic changes (pathogenic germline variants—PGV) is therefore of great importance in predicting the risk of lung cancer. It has been established that it occurs in a fairly large group, 15% of patients with lung cancer. This is also important information for the closest relatives of these patients. Early detection of genetic predisposition allows one to reduce the risk of this disease, as well as detect it early, when even more effective treatment is possible [84]. The study included 7788 patients with lung cancer, among whom 1161 patients were found to have inherited genetic changes in 81 known cancer mutations so far. Approximately 95.1% of patients with these lesions could be treated with available therapies or could be covered by early detection of the disease [84].

Table 6 below presents the risk factors for lung cancer.

## 9. Smoking

**Figure 3:**
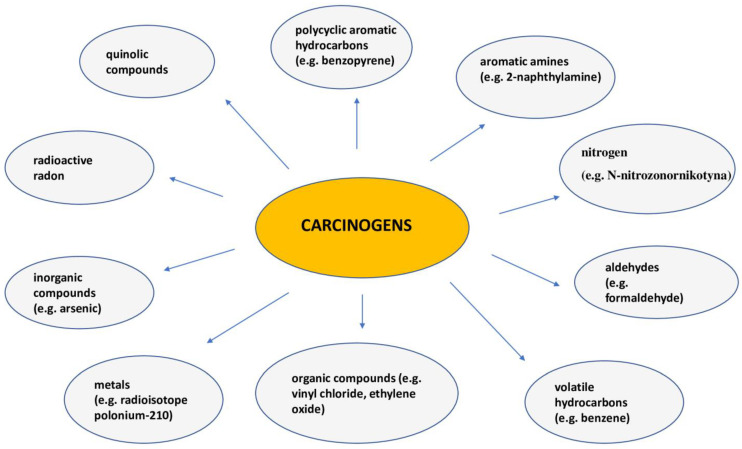
Carcinogens contained in cigarette smoke.

Literature data clearly show that smoking is currently one of the main carcinogens affecting the shortening of life expectancy [91,92,93]. This is confirmed by the calculations of special medical calculators, which show that smoking one cigarette shortens life by about 11 min on average, and this translates into about 13.2 years shorter survival in men and 14.5 years in women compared to people who have never smoked [94,95,96]. Smoking is responsible for about 90% of lung cancer deaths in men and 75–80% of lung cancer deaths in women. The risk of developing the disease depends, among other things, on the number of cigarettes smoked per day and the duration of the addiction [23]. Unfortunately, environmental exposure to tobacco smoke is one of the most common causes of lung cancer in non-smokers, as confirmed by epidemiological data conducted since 1981 [37]. Smoking is also associated with a higher risk of developing other cancers, e.g., of the oral cavity, throat, esophagus, stomach, pancreas, bladder, kidney and cervix, as well as the hematopoietic system [97]. Spectral analysis of cigarette smoke showed that it contains over 6000 compounds, of which over 60 are carcinogens [98]. Figure 3 shows the most important of them.

The above-mentioned compounds have the ability to bind to DNA, which results in their carcinogenic effect, and this is associated with the induction of the formation of further mutations [99,100]. In the literature, the observation that nicotine has the ability to inhibit the process of apoptosis is emphasized, and this ensures the “immortality” of the cell, especially in the course of anticancer therapy with cytostatics. In addition, the components of tobacco smoke have a particularly important immunosuppressive effect by inhibiting both specific and non-specific immune responses, which promotes the formation of cancer by weakening the body’s natural defense mechanisms [101,102]. Other mechanisms linking smoking with cancer formation, apart from the most important mutagenic effect or activation of pathways associated with proliferation, are epigenetic disorders. Hypomethylation of promoter regions of tumor suppressor genes leads to excessive proliferation [103,104,105].

Smoking can affect the immune system in various ways, intensifying inflammatory allergic and autoimmune reactions or reducing systemic activity towards infections [106,107]. Table 7 shows the effects of smoking and nicotine exposure on immune function.

## 10. Treatment of Patients with Non-Small Cell Lung Cancer

Treatment of patients with non-small cell lung cancer depends on the stage of the tumor and is divided into three clinical situations: early (stage I–II), locally advanced (stage III) and metastatic (stage IV) [108,109].

### 10.1. Surgical Treatment of Patients with Non-Small Cell Lung Cancer

**Figure 4:**
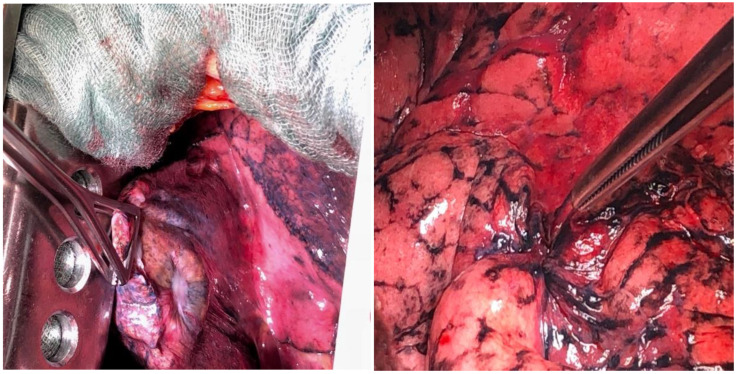
Intraoperative image of non-small cell lung carcinoma infiltrating the pulmonary pleura of a patient treated surgically at the Clinic of Thoracic Surgery and Respiratory Rehabilitation, Medical University of Lodz, Regional Multi-Specialist Center for Oncology and Traumatology of the Nicolaus Copernicus Memorial Hospital in Lodz, Lodz, Poland.

In patients with stage I and II NSCLC and in selected patients with stage IIIA (without the N2 feature), the treatment of choice is surgery (Figure 4). This type of treatment can only be offered to about 20% of patients. The gold standard is to carry out full diagnostics, both radiological and, in doubtful cases, endoscopic (EBUS-TBNA, EUS-FNA, mediastinoscopy) before the planned surgery [110,111,112,113]. This is very important because in patients with stage IIIA metastases to the N2 lymph nodes, the results of primary surgical treatment are still unsatisfactory [114].

**Figure 5:**
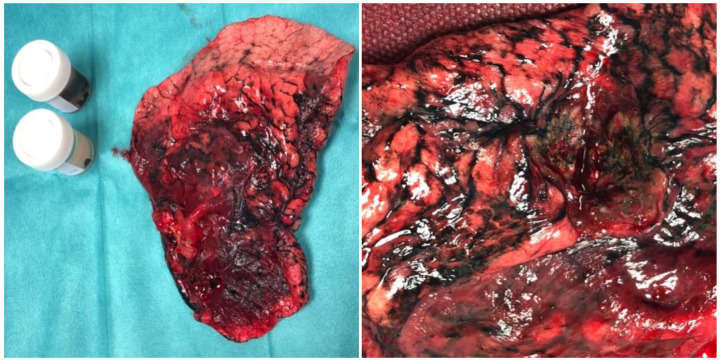
Postoperative material—upper lobe of the left lung and mediastinal lymph nodes of a patient treated surgically at the Clinic of Thoracic Surgery and Respiratory Rehabilitation, Medical University of Lodz, Regional Multi-Specialist Center for Oncology and Traumatology of the Nicolaus Copernicus Memorial Hospital in Lodz, Lodz, Poland.

The classic surgical procedure involves performing a complete resection of the lung parenchyma in which the tumor is present. The method of choice for patients who qualify for surgery is lobectomy (Figure 5) [115]. A resection that is more limited than lobectomy, segmentectomy or the so-called wedge resection, is justified only in patients with significant limitation of respiratory reserves. Pneumonectomy is generally performed only when the lobectomy does not ensure complete excision. All types of resection must be accompanied by the removal of the hilar and mediastinal lymph nodes. Postoperative material should contain at least six lymph nodes from groups N1 (three nodes) and N2 (three nodes) [116].

The effect of lymphadenectomy extent on the outcomes of surgical treatment has not been definitively established, but a more extensive excision of the lymphadenectomy system allows for a more complete determination of the postoperative stage of the tumor and facilitates qualification for adjuvant treatment [117]. In patients with stage I cancer and a tumor with a diameter of less than 5 cm, the lobectomy procedure can be performed using the video-assisted thoracoscopic surgery method. The above method consists of anatomical excision of the lung lobe with the tumor and mediastinal lymph nodes with a camera and specially adapted tools through a small 4–6 cm incision [118]. The main advantage of the above method is the reduction in pain after the procedure, as well as the reduction in the occurrence of postoperative complications, especially pulmonary complications, compared to the procedure of classic lobectomy, performed by thoracotomy. Such a procedure is also associated with a reduction in both the time of pleural drainage and the length of the patient’s hospitalization [119].

### 10.2. Treatment of Patients with Early (I–II) and Locally Advanced (III) Non-Small Cell Lung Cancer

It is advisable to use adjuvant chemotherapy using regimens based on cisplatin and vinorelbine.

As a standard, adjuvant treatment includes three to four cycles of chemotherapy and is used in patients at stages II–III. Such management improves long-term survival rates by about five percentage points [120,121]. Postoperative radiotherapy after radical surgery worsens the prognosis, which is why it is not currently recommended according to the standards of oncological management [122]. Postoperative radiotherapy, on the other hand, is recommended in cases where the procedure was microscopically non-radical (R1), i.e., tumor cells in the incision line were confirmed in the pathomorphological postoperative examination. The second indication for adjuvant radiotherapy is the unreliability of the postoperative determination of the condition of the mediastinal lymph nodes [123]. The results of preoperative chemotherapy are contradictory, and such treatment in patients with resectable lung cancer is not recommended [124]. The standard of care in patients with locally advanced lung cancer is the use of cytostatic therapy preceding stage IIIa surgery or combined with radical radiotherapy (synchronously or sequentially; most patients at stage IIIA and some at stage IIIB) [109]. Synchronous treatment increases local control and improves overall survival by five percentage points, at the expense of increased toxicity of therapy [125]. The phase III PACIFIC study demonstrated the importance of consolidation treatment with the use of an antibody targeting anti-PD-L1 immune checkpoints—durvalumab—in a population of 713 patients. One-year consolidation treatment after two cycles of concomitant radiochemotherapy increases progression-free survival and overall survival [126,127].

### 10.3. Treatment of Patients with Advanced Stage (IV) Non-Small Cell Lung Cancer

In stage IV non-small cell lung cancer, palliative chemotherapy is the dominant form of therapy, giving a 20–30% objective response rate [128,129]. The most commonly used cystostat was and is cisplatin in combination with gemcitabine, vinorelbine, paclitaxel or docetaxel [130]. The ECOG study from 1994 showed equal efficacy in both survival and response to treatment regardless of the non-platinum component of the chemotherapy regimen. In the treatment of advanced disease, cisplatin is more active than carboplatin—with a higher objective response rate (ORR: 30 vs. 24%) [131]. For many years, therapeutic decisions were influenced only by the pathomorphological division into small and non-small cell carcinoma. Histological types (glandular, squamous and large cell) did not determine the method of systemic treatment [132].

The first step towards personalized treatment was the study by Scagliotti et al., which showed that the effectiveness of chemotherapy may depend on the histological type. Patients diagnosed with non-squamous cell carcinoma receiving cisplatin chemotherapy in combination with pemetrexed had a 1.7-month higher median OS compared to cisplatin doublet therapy with gemcitabine. On the contrary, in patients diagnosed with squamous cell carcinoma, treatment with cisplatin and gemcitabine was more effective [133]. In patients diagnosed with adenocarcinoma in whom the presence of an EGFR mutation suggestive of tyrosine kinase inhibitor activity of this receptor has been confirmed, the use of EGFR TKIs—erlotinib, gefytinib or afatinib—as early as in the first line of palliative therapy is indicated [134]. If the disease progresses during treatment with these TKIs and the T790M mutation in exon 20 is detected, another EGRF kinase inhibitor, osimertinib, may be be more effective than chemotherapy [135,136]. Crizotinib is the first ALK kinase inhibitor with activity greater than chemotherapy in phase I and subsequent lines of palliative treatment in patients with ALK-rearranged NSCLC [137]. In the case of resistance to crizotinib therapy, other ALK inhibitors—ceritinib and alectinib—were found to be effective—and were approved due to the positive results of two phase II studies [129]. Hopes for improving the outcomes of first-line palliative treatment of NSCLC have been brought by studies using immune checkpoint inhibitors. Therapy with pembrolizumab (an anti-PD1 antibody) in patients with high PD-L1 expression (on at least 50% of cancer cells) is associated not only with an extension of the free time to progression but also with a significant improvement in prognosis. Unfortunately, the benefit of such therapy is limited to about 20% of patients with NSCLC (high expression of PD-L1 is found in about 30% of cases) [138,139,140,141]. For several years, the possibilities of using immunotherapy after the failure of first-line chemotherapy have been known. The use of anti-PD1 antibodies, such as nivolumab and pembrolizumab, as well as anti-PD-L1—atezolizumab, has a positive effect on survival, but direct responses to treatment are observed in only 20% of patients with NSCLC [142,143,144].

## 11. Limitations

The poor prognosis of people diagnosed with lung cancer is a challenge worldwide. A chance to achieve a better situation should be sought in more effective primary prevention (further reduction in exposure to harmful products of tobacco combustion), early detection and efficient determination of pathomorphological advancement and diagnosis, increasing the percentage of patients undergoing complete surgical treatment, expanding the possibilities of using modern methods of radiotherapy and radiochemotherapy and rationalization of systemic treatment. Rationalization of systemic treatment includes the correctness of the use of chemotherapy (e.g., qualifying patients with real chances of obtaining benefits or adjusting drugs to the histological type) and the use—in justified clinical situations—of available targeted drugs. There are many challenges in addressing the risks associated with lung cancer. Efforts should be made to create centers with full diagnostic and therapeutic capabilities (the so-called centers of excellence). It is necessary to increase the rate of complete resections of the lung parenchyma following an earlier diagnosis of lung cancer. It is also necessary to shorten the waiting time for pathomorphological examinations and increase the share of radiotherapy (especially combined management with radiotherapy and chemotherapy). The use of systemic treatment should be more rational, which means proper qualification and management of chemotherapy and increasing the possibility of treatment aimed at molecular targets. The last of the mentioned problems is related—primarily—to the improper organization of molecular predictive factor testing (insufficient number of laboratories performing tests in accordance with standards and an inadequate system of financing molecular diagnostics). An important element is to increase the possibility of patients participating in controlled clinical trials on new treatment methods and to use more modern methods of determining the value of new drugs. It is also extremely important to provide proper care for patients after treatment and early detection of recurrences and complications.

## 12. Conclusions

Risk factors for lung cancer have been mostly understood and well characterized. Primary prevention of this disease therefore seems to be easy to implement by eliminating environmental hazards and smoking. Despite this, lung cancer remains the leading cause of death among malignant cancers in all highly developed countries. The causes of this phenomenon should be sought in the growing problem of environmental pollution, but above all in the difficulty of eliminating the addiction to smoking. In the prevention of lung cancer, the basic factor is not smoking. Tobacco smoke is the most common cause of lung cancer. It is worth noting that electronic cigarettes are also not recommended in the context of prevention. The lack of proper education means that young people continue to reach for nicotine-containing products, first e-cigarettes and then traditional cigarettes. However, nicotine addiction is extremely strong in many people, and eliminating the addiction using traditional methods (psychotherapy, nicotine replacement therapy, pharmacotherapy) turns out to be impossible. In such cases, reducing the health risk associated with smoking cigarettes can be achieved by replacing them with smokeless products containing nicotine. Many scientific studies have shown that aerosols from e-cigarettes and tobacco heating devices contain over 90% less carcinogenic substances than cigarette smoke [145].

However, it should be remembered that while the composition of the aerosol is known in tobacco heating devices, in the case of e-liquids, it can be modified by the owners of e-cigarettes or the companies producing them (this has been the cause of many cases of acute lung injury in people using e-liquids containing THC and vitamin E acetate). Therefore, in many countries (USA, the Netherlands, Belgium, Germany), HnB devices have been defined as products with a reduced health risk compared to traditional cigarettes, and international experts issue cautious recommendations on the possibility of reducing the health risk in cigarette smokers by replacing them with tobacco heating products [146].

It has been proven that proper nutrition, with particular emphasis on vegetables and fruits, as well as regular physical activity have a positive effect on reducing the risk of lung cancer [147].

Preventive programs are also being carried out for people who are potentially at risk of developing this type of cancer. The screening test involves performing a low-dose computed tomography scan to detect lung cancer at an early stage [148].

To sum up, the vast majority of lung cancer cases are the result of inhalation of substances that promote their formation, e.g., tobacco smoke, car exhaust fumes and smoke from burning coal. Proper prevention of lung cancer and reduction in exposure to carcinogens increase the chance of staying healthy.

## Figures and Tables

**Table 1:** Local and general symptoms occurring in the course of lung cancer [3].

Local Symptoms of Lung Cancer	General Symptoms of Lung Cancer
cough (especially a change in its character)	arthralgia
dyspnea	general weakness
hemoptysis	weight loss
chest pain	weight gain
recurrent pneumonia	superficial sensory disorders
hoarseness	symptoms of thrombophlebitis
swallowing disorder	symptoms of paraneoplastic syndromes: wasting syndrome exhaustion syndrome syndrome of inappropriate antidiuretic hormone secretion (SIADH) Cushing’s syndrome hypercalcemia Lambert–Eaton syndrome retinopathy encephalopathy
shoulder pain	
superior vena cava syndrome	
Horner’s syndrome	

**Table 2:** Tests performed as part of the staging of lung cancer.

Primary Tumor Assessment	Lymph Node Assessment	Assessment of Distant Organs
X-ray CT (less often MRI) Bronchofiberoscopy Transbronchial biopsy (“blind”, “semi-blind” transbronchial biopsy using radial ultrasound transducer, endobronchial ultrasonography, esophageal ultrasonography) Chest wall biopsy (peripheral lesions) Cryobiopsy of peripheral lesions Pap smear examination of pleural or pericardial fluid	Computed tomography (less often magnetic resonance) Bronchofiberoscopy Mediastinoscopy Parasternal medistinotomy Positron emission tomography—computed tomography Physical examination Fine needle aspiration biopsy or surgical biopsy of suspected supraclavicular nodes Thoracoscopy Esophageal ultrasonography Endobronchial ultrasonography	Abdominal ultrasound or computed tomography Biopsy of a single focus in the adrenal gland with suspected metastasis Computed tomography or magnetic resonance imaging of the brain [SCLC—always; NSCLC—before planned radical treatment (see the text for details) and in case of clinical suspicion] Bone scintigraphy (SCLC—planned combination therapy; NSCLC—Clinical Suspicion) Positron emission tomography—computed tomography Fine needle aspiration biopsy or surgical biopsy of suspicious lesions

**Table 3:** Classification of TNM in lung cancer (UICC, 2016) [44].

Feature	Characteristics
T	
TX	The primary tumor cannot be evaluated or its presence has been demonstrated only on the basis of the presence of tumor cells in the bronchial secretion, without the possibility of visualization by imaging and bronchoscopy
T0	Absent of primary tumor features
Tis	carcinoma in situ
T1	Tumor with a diameter of not more than 3 cm, surrounded by pulmonary parenchyma or pulmonary pleura, without infiltration of the main bronchi
T1a(mi)	Minimally invasive adenocarcinoma—single tumor—adenocarcinoma ≤ 3 cm, with a predominantly lepidic growth type, with an invasive component ≤ 5 mm in the largest dimension
T1a	Tumor with the largest size of 1 cm (also a rare primary tumor spreading superficially, of any dimension, the invasive component of which is limited to the bronchial wall, even if it occurs in the main bronchi)
T1b	Tumor with the largest size exceeding 1 cm but not more than 2 cm
T1c	Tumor with the largest size exceeding 2 cm but not more than 3 cm
T2	A tumor with a diameter of more than 3 cm but not more than 5 cm, or a tumor with at least one of the following: - main bronchial involvement without main spur involvement - visceral pleura infiltration - concomitant atelectasis or pneumonia extending to the hilar area, involving both part and all of the lung
T2a	Tumor with a diameter of more than 3 cm but not more than 4 cm
T2b	Tumor with a diameter of more than 4 cm but not more than 5 cm
T3	A tumor with a diameter of more than 5 cm but not more than 7 cm, or a tumor of any size with the presence of infiltration of one of the following areas: - chest wall (including tumor of the upper thoracic opening) - phrenic nerve - wall pericardium or Tumor with co-occurrence of satellite lesions in the same lobe of the lung
T4	A tumor with a diameter of more than 7 cm or a tumor of any size with the presence of infiltration of one of the following areas: - mediastinum - diaphragm - heart - large dishes - trachea - recurrent laryngeal nerve - esophagus - circles - main spur or Tumor of any size with co-occurrence of satellite lesions in another lobe of the same lung
N	
NX	Inability to assess the surrounding lymph nodes
N0	Absence of metastases in the surrounding lymph nodes
N1	Metastases in peribronchial and/or hilar lymph nodes on the side of the primary tumor and intrapulmonary (including direct involvement by continuity from the side of the primary tumor)
N2	Metastases in the mediastinal lymph nodes on the side of the primary tumor and/or bifurcation of the trachea
N3	Metastases in the mediastinal lymph nodes or hilum on the contralateral side, under the inclined muscle and/or supraclavicular on the side of the primary tumor or on the opposite side
M	
MX	Inability to assess metastases to distant organs
M0	Absence of distant metastases
M1	Presence of distant metastases
M1a	Satellite lesions in the opposite lung, presence of pleura/pericardial nodules or presence of tumor cells in the pleura/pericardial fluid
M1b	Presence of a single distant metastasis in one organ
M1c	Multiple metastases in one organ or metastases in different organs

**Table 4:** Stages of lung cancer (UICC, 2016) [44].

Stages	Characteristics		
Occult cancer	TX	N0	M0
0	Tis	N0	M0
IA1	T1a(mi), T1a	N0	M0
IA2	T1b	N0	M0
IA3	T1c	N0	M0
IB	T2a	N0	M0
IIA	T2b	N0	M0
IIB	T1a, T1b, T1c T2a, T2b T3	N1 N1 N0	M0 M0 M0
IIIA	T1a, T1b, T1c, T2a, T2b T3 T4	N2 N2 N1 N0, N1	M0 M0 M0 M0
IIIB	T3, T4 T1a, T1b, T1c, T2a, T2b	N2 N3 N3	M0 M0 M0
IIIC	T3, T4	N3	M0
IVA	each T	each N	M1a, M1b
IVB	each T	each N	M1c

**Table 5:** Pathomorphological classification of lung cancer according to the World Health Organization from 2015 [62].

Type	Subtype
Adnocarcinoma	wallpapering adenocarcinoma (lepidic adenocarcinoma) acinar adenocarcinoma (acinar adenocarcinoma) papillary adenocarcinoma (papillary adenocarcinoma) small-papillary adenocarcinoma (micropapillary adenocarcinoma) solid adenocarcinoma invasive mucinous adenocarcinoma with mixed mucinous and nonmucinous carcinoma colloid adenocarcinoma fetal adenocarcinoma enteric-type adenocarcinoma minimally invasive adenocarcinoma with carcinoma with mucinous or nonmucinous carcinoma pre-invasive lesions — atypical adenomatous hyperplasia — adenocarcinoma in situ with or without mucus production (mucinous lub nonmucinous)
Squamous cell carcinoma	keratinizing squamous-cell carcinoma non-keratinizing squamous-cell carcinoma squamous-cell carcinoma in situ
Neuroendocrine tumors	small-cell carcinoma with combined carcinoma large-cell carcinoma with combined carcinoma typical and atypical carcinoids diffuse idiopathic pulmonary neuroendocrine hyperplasia
Large cell carcinoma	
Adenoid squamous cell carcinoma	
Sarcoma cankers	pleomorphic sarcomatoid carcinoma spindle-cell sarcomatoid carcinoma giant-cell sarcomatoid carcinoma carcinosarcoma pulmonary blastoma
Salivary gland type carcinomas	mucoepidermoid carcinoma adenoid-cystic carcinoma
Unclassified	

**Table 6:** The risk factors for lung cancer.

Risk Factor for Lung Cancer	
smoking	Smoking is the cause of 90% of lung cancer cases in men and 80% in women. Smokers have a 30 times higher risk of death from lung cancer than non-smokers. Cigarette smoke hides over 7000 chemical compounds, including over 70 compounds considered carcinogenic. Secondhand smoke is also associated with a higher risk of lung cancer compared to people who are not exposed to tobacco smoke. It is estimated that about 20–50% of “non-smokers” who suffer from lung cancer are passive smokers [85].
alcohol	Studies indicate that people who abused alcohol were more likely to develop lung cancer. Researchers do not provide exact data but estimate that it may be related to another factor: smoking. Studies show that people are more likely to reach for cigarettes when they drink. Researchers at the University of Liverpool studied 125,249 British drinkers and 47,967 Americans. As many as six genes have been identified that, in their opinion, are associated with excessive alcohol consumption and, consequently, with lung cancer [86].
genetic predisposition	The role of genetic factors is still quite poorly understood. The high incidence of lung cancer in some families is associated with a genetically determined tendency to overactivate carcinogenic compounds contained in tobacco smoke or to remove these compounds from the body too slowly. A tendency to slowly repair DNA damage in respiratory epithelial cells after the action of carcinogens is also inherited. To sum up, it can be stated that the hereditary condition is primarily a special susceptibility to the carcinogenic effects of tobacco. This inheritance is the result of the presence of polymorphisms (population variants) in many genes, and there are currently no reliable genetic tests to determine the high risk of developing lung cancer. Research by American specialists shows that the detection of inherited genetic changes (pathogenic germline variants—PGV) is of great importance in predicting the risk of lung cancer. It has been established that it occurs in a fairly large group, i.e., 15% of patients with lung cancer [84].
occupational factors	Exposure to many occupational factors has consequences in the form of the development of lung diseases, including lung cancer. The most important occupational carcinogens include asbestos, silica, heavy metals and polycyclic aromatic hydrocarbons [87]. All forms of asbestos (chrysotile and amphiboles, including crocidolite, amosite and tremolite) are carcinogenic, although the potency of chrysotile is less than that of other types, likely due to its more effective removal from the lungs. In many underdeveloped countries, occupational exposure to asbestos remains widespread [87,88]. Elevated risk of lung cancer has been reported in several industries and occupations associated with exposure to polycyclic aromatic hydrocarbons, such as aluminum production, coal gasification, coke production, iron and steel foundries, tar distillation, roofing and chimney cleaning. It has also been suggested that people employed in several other industries have increased risk of lung cancer, including shale oil mining, wood impregnation, roofing and carbon electrode manufacturing [88].
environmental factors	Air pollution data show that lung cancer incidence increases by 30–50% in areas with high levels of ambient air pollution compared to areas with lower levels [89,90]. Many studies carried out so far clearly show that the risk of developing lung cancer is much higher in highly urbanized, industrialized regions with a developed transport network, in particular based on the use of internal combustion engines [37].
age	The risk of developing lung cancer also increases with age. The majority of lung cancers occur after the age of 50 (96% of cases in men and 95% of cases in women), with about 50% of cases in both sexes occurring in the population over 65 years of age. The risk of developing lung cancer peaks in men in the eighth decade of life and in women at the turn of the sixth and seventh decades of life [25].

**Table 7:** Effects of smoking and nicotine exposure on immune function.

Immunosuppressive Effects	Pro-Inflammatory Effects
Effects on dendritic cells and their ability to present antigen Suppression of dendritic cell maturation and cytokine release	Activation of acquired immunity with the involvement of dendritic cells
Effects on neutrophils and macrophages Suppression of neutrophil-mediated inflammatory effects Reduction in neutrophil migration and chemotaxis Reduction in macrophage activity towards intracellular organisms	Increase in neutrophil levels in circulation
Effects on the T cell population Nicotine inhibits the cellular response associated with the formation of antibodies, interferes with antigen-mediated signaling in T lymphocytes, induces T cell anergy	Polyphenol-rich glycoprotein stimulates peripheral T cell proliferation Increase in the number of circulating T cells Abnormal CD4(+)/CD8(+) ratio Tilting of activity towards the sensitization pathway involving Th2 lymphocytes
Effects on B cell populations	
	Increase in autoreactive B lymphocytes
Effect on humoral response Circulating immunoglobulin reduction	
Effects on inflammatory markers and mediators Zahamowanie uwalniania IL-1, IL-2, IL-10, TNF-α i IFN-γ Inhibition of IL-8 release by endothelial cells	Chronic smoking leads to an increase in the concentration of acute phase proteins and pro-inflammatory cytokines, especially TNF-α, TNF-α and IL-6 receptors
Other general non-specific mechanisms: Attenuation of IFN signaling	Exposure and release of autoantibodies Release of intracellular antigens due to necrosis induced by tissue hypoxia or toxins
	Increase in the concentration of free radicals that interact with DNA
